# Association between time perspective and organic food consumption in a large sample of adults

**DOI:** 10.1186/s12937-017-0311-0

**Published:** 2018-01-05

**Authors:** Marc Bénard, Julia Baudry, Caroline Méjean, Denis Lairon, Kelly Virecoulon Giudici, Fabrice Etilé, Gérard Reach, Serge Hercberg, Emmanuelle Kesse-Guyot, Sandrine Péneau

**Affiliations:** 1 Equipe de Recherche en Epidémiologie Nutritionnelle, Centre de Recherche en Epidémiologie et Statistique Sorbonne Paris Cité, INSERM U1153, INRA U1125, Cnam, Université Paris 13, 74, rue Marcel Cachin, 93017 Bobigny, France; 2 INRA, UMR1110 MOISA, F-34000 Montpellier, France; 3 Aix Marseille Université, Nutrition Obésité et Risque Thrombotique (NORT), INSERM UMR S 1062, INRA 1260, Marseille, France; 4 Paris School of Economics and INRA, UMR1393 PjSE, 48 Boulevard Jourdan, 75014 Paris, France; 5 Service d’Endocrinologie, Diabétologie, Maladies Métaboliques, Hôpital Avicenne, Bobigny, France; 6 Unité de Surveillance en Epidémiologie Nutritionnelle, Institut de Veille Sanitaire, Université Paris 13, Bobigny, France; 7 Département de Santé Publique, Hôpital Avicenne, Bobigny, France

**Keywords:** Organic food consumption, Nutrition, Consideration of future consequences, Psychology

## Abstract

**Background:**

Organic food intake has risen in many countries during the past decades. Even though motivations associated with such choice have been studied, psychological traits preceding these motivations have rarely been explored. Consideration of future consequences (CFC) represents the extent to which individuals consider future versus immediate consequences of their current behaviors. Consequently, a future oriented personality may be an important characteristic of organic food consumers. The objective was to analyze the association between CFC and organic food consumption in a large sample of the adult general population.

**Methods:**

In 2014, a sample of 27,634 participants from the NutriNet-Santé cohort study completed the CFC questionnaire and an Organic-Food Frequency questionnaire. For each food group (17 groups), non-organic food consumers were compared to organic food consumers across quartiles of the CFC using multiple logistic regressions. Moreover, adjusted means of proportions of organic food intakes out of total food intakes were compared between quartiles of the CFC. Analyses were adjusted for socio-demographic, lifestyle and dietary characteristics.

**Results:**

Participants with higher CFC were more likely to consume organic food (OR quartile 4 (Q4) vs. Q1 = 1.88, 95% CI: 1.62, 2.20). Overall, future oriented participants were more likely to consume 14 food groups. The strongest associations were observed for starchy refined foods (OR = 1.78, 95% CI: 1.63, 1.94), and fruits and vegetables (OR = 1.74, 95% CI: 1.58, 1.92). The contribution of organic food intake out of total food intake was 33% higher in the Q4 compared to Q1. More precisely, the contribution of organic food consumed was higher in the Q4 for 16 food groups. The highest relative differences between Q4 and Q1 were observed for starchy refined foods (22%) and non-alcoholic beverages (21%). Seafood was the only food group without a significant difference.

**Conclusions:**

This study provides information on the personality of organic food consumers in a large sample of adult participants. Consideration of future consequences could represent a significant psychological determinant of organic food consumption.

**Electronic supplementary material:**

The online version of this article (10.1186/s12937-017-0311-0) contains supplementary material, which is available to authorized users.

## Background

The demand of organic foods has been notably increasing over the past years. In France, sales of organic products represented 5.5 billion euros in 2014 corresponding to a 10% increase from the previous year as it was the case in most European countries [1]. The major reasons regarding the purchase of organic foods include altruistic motives such as environmental and ethics aspects [2–7], and self-centered motives such as health, food safety, and sensory aspects [2, 4, 6, 8, 9]. Despite individual differences concerning these motivations, specific psychological traits which could lead to these motivations, and thus to an organic food consumption oriented behavior, have rarely been explored.

Consideration of Future Consequences (CFC) is a construct which measures “the extent to which individuals consider the potential distant outcomes of their current behaviors and the extent by which they are influenced by these potential outcomes [10].” Individuals with a low CFC are expected to act on their immediate needs and concerns whereas individuals with a high CFC are expected to consider the future implication of their behavior and to use their distant goals as guides for their current actions. Higher CFC have been shown to be associated with healthy and environmentally friendly behaviors [10]. In particular, several studies found that future oriented individuals were more likely to have a health oriented behavior, such as exercising more [11, 12], presenting healthy eating attitudes and intentions [13], being more sensitive to health communications to get tested for colorectal cancer [14], and participating in diabetes screening [15]. A meta-analysis measuring time perspective with different constructs found that future time perspective influenced individual attitudes and behaviors towards the environment [16], such as environmental preservation [17], recycling and waste reduction [18], and water conservation [19]. Finally, a study reported future oriented consumers to be more careful about organic labels, suggesting a higher interest in health related and sustainability issues [20]. However, to our knowledge no data are available on the relationship between time orientation and organic food intakes.

The aim of this study was therefore to analyze the association between consideration of futures consequences and the consumption of 17 organic food groups in a sample of the general population participating in the NutriNet-Santé cohort study by taking into account sociodemographic, lifestyle and dietary characteristics. Firstly, we wanted to assess whether organic food consumers were more likely to be future oriented compared to nonorganic food consumers. Then, we quantitatively analyzed intakes of organic food according to the individual level of consideration of future consequences.

## Methods

### Population

This study was conducted as part of the NutriNet-Santé study, which is a large ongoing web-based prospective cohort started in France in May 2009.The rationale, design and methods of the study have been described elsewhere [21]. Its overall aim is to explore the relationships between nutrition and health and the determinants of eating behavior and nutritional status. Participants are adult volunteers (age ≥ 18 years) of the general French population with a scheduled follow-up of at least 10 years. At inclusion, participants have to complete several self-reported web-based questionnaires to assess their diet, their physical activity, anthropometric measures, lifestyle characteristics, socioeconomic conditions and health status. Participants complete this set of questionnaires every year after inclusion. Finally, another set of optional questionnaires related to determinants of eating behaviors, nutritional status, and specific aspects related to health are sent to every participant each month. A flowchart of the participants included in this study is available as Additional file 1.

This study was conducted in accordance with the guidelines of the Declaration of Helsinki, and all procedures were approved by the International Research Board of the French Institute for Health and Medical Research (IRB Inserm n° 0000388FWA00005831) and the Commission Nationale Informatique et Libertés (CNIL n° 908450 and n° 909216). Electronic informed consent was obtained from all participants.

### Data collection

#### Consideration of future consequences

Consideration of Future Consequences was assessed with the French version of the CFC-12 questionnaire [22] over a 6-month period from June to November 2014. The CFC-12 is a 12-item self-report questionnaire [10] developed to measure the extent to which individuals consider distant versus immediate consequences of their behavior. Each item is measured on a 5-point Likert scale ranging from “extremely uncharacteristic” to “extremely characteristic”. An example of the items of the CFC-12 is as followed: *I consider how things might be in the future, and try to influence those things with my day to day behavior*. The total score is obtained by summing each item ratings leading to a possible range from 12 to 60 (higher scores indicating greater consideration of future consequences). Participants were divided into 4 categories determined by quartiles of the total score (Q1, Q2, Q3, and Q4). A good internal consistency was obtained in our sample with a Cronbach’s α of 0.79.

#### Dietary intake

To assess their organic food consumption, participants completed a semi-quantitative organic food frequency questionnaire (Org-FFQ) by providing the frequency and portion sizes of consumed foods and beverages. The Org-FFQ was administered over a 5-month period from June to October 2014. This questionnaire was based on a validated food frequency questionnaire [23] supplemented by a section pertaining to the frequency of organic food consumption. More precisely, participants were asked to report their frequency of consumption and the quantity consumed over the past year for 264 items allowing to assess total food intakes (g/d). In addition, the frequency of organic food consumption for each item was assessed with a 5-point Likert scale ranging from never to always. Organic food intake (g/d) was obtained for each item by applying a weight of 0, 0.25, 0.5, 0.75 and 1 to the five respective categories of frequency (never, rarely, half the time, often and always). A full description of the Org-FFQ as well as sensitivity analyses pertaining to weighting can be found elsewhere [24].

Beverage and food items were aggregated into 17 food groups: fruits and vegetables (including juices and soups); seafood; meat, poultry and processed meat; eggs; dairy products; starchy refined foods; whole-grain products; legumes; fats (oil, butter, and margarine); fatty sweets (including cake, chocolate, ice cream, and pancakes); non-fatty sweets (including honey, jelly, sugar, and candy); alcoholic beverages; non-alcoholic beverages; fast food; snacks (including chips and salted biscuits); dressings and sauces; and dairy products and meat substitutes (including soya-based products). For each food group, contribution of organic food consumed was estimated by computing the organic food intake of the food group (g/d) out of the total food intake of the food group (g/d) multiplied by 100. Total energy intake (kcal/day) was also calculated using a validated composition table [25]. Participants with unlikely estimates of energy intake were identified as under- and over-reporting participants against estimated energy requirement. Basal metabolic rate (BMR) was calculated according to age, gender, weight and height using Schofield’s equations [26]. The ratio between energy intake and estimated energy requirement (physical activity level x BMR, with physical activity level set by default at 1.55) was calculated and individuals with ratios below the 1st percentile (0.35) or above the 99th percentile (1.93) were excluded. These cutoffs were calculated on the validated FFQ for usual dietary intake used in the NutriNet-Santé cohort [23].

#### Socio-demographic, economic, anthropometric and lifestyle characteristics

Potential confounders of the relationship between CFC and organic food consumption were collected based on information provided yearly by the participants after their inclusion: age (years), gender, education level (primary, secondary, undergraduate, and postgraduate), occupational status (unemployed, student, self-employed and farmer, employee and manual worker, managerial staff and intellectual profession, intermediate profession, and retired), monthly income per household unit, place of residence (rural community, urban unit with a population < 20,000 inhabitants, urban unit with a population between 20,000 and 200,000 inhabitants, and urban unit with a population > 200,000 inhabitants), and BMI (kg/m^2^). More precisely, monthly income per household unit was calculated with information about income and composition. The number of people of the household was converted into a number of consumption units (CU) according to a weighting system: one CU is attributed for the first adult in the household, 0.5 for other persons aged 14 or older and 0.3 for children under 14 [27]. Categories of income were defined as followed: < 1200; 1200–1799; 1800–2299; 2300–2699; 2700–3699; and > 3700 euros per household unit as well as “unwilling to answer”.

The Programme National Nutrition Santé Guidelines Score (PNNS-GS), which is an a priori nutritional diet quality score reflecting the adherence to the French nutritional recommendations of the participants [28], was considered as a confounder in the analyses. The original score includes 13 components: eight refer to food serving recommendations, four refer to moderation of nutrients or food, and one refers to physical activity. Points are deducted for overconsumption of salt and sweets. Points are also deducted from the total when energy intake exceeds the energy needs by more than 5%. A modified version of the PNNS-GS (mPNNS-GS) that did not include the physical activity component was used in this study. The score has a range of 0 to 13.5 points, with a higher score indicating a better overall nutritional quality of the diet.

### Statistical analysis

The characteristics of the sample across quartiles of the CFC-12 were compared with linear contrast tests for continuous variables, and with Mantel-Haenszel chi-square tests for categorical variables. Logistic regression models were performed between organic food consumption as a dependent variable (organic food consumer versus non-organic food consumer (reference)) for each of the 17 food groups) and the four categories (quartile, Q) of the CFC-12 as the main independent variable (Q1 as reference). The strength of the association was estimated by calculating odds ratios (ORs) and 95% confidence intervals (95% CI). Furthermore, adjusted means of proportions of the contribution of organic food to the total food intake by food group were compared across categories of the CFC-12 for the 17 food groups among organic food consumers only. A percentage of the relative difference between adjusted means of Q4 and Q1 was calculated to estimate the effect size of the differences. For every analysis on each food group, participants who did not report to consume at least one food item of the group (organic or non-organic food intakes) were excluded from the analysis of this food group. Since socio-economic positions are associated with CFC [29] and dietary intakes, all adjusted models included the following confounders: age, gender, education level, occupational status, monthly income per household unit, and place of residence. In addition, it has been suggested that time perspective can predict or be predicted by health behaviors [12]. Moreover, BMI, energy intake, mPNNS-GS (diet quality), and total food intake of the food group all predict the level of organic food consumption and were thus taken into account. No significant interaction terms were found between the CFC-12 and confounders. Missing data on confounding variables were handled with multiple imputation by chained eqs. (20 imputed datasets) [30].

All tests of statistical significance were 2-sided and significance was set at 5%. A Hochberg procedure was applied to correct for multiple testing. Statistical analyses were performed using SAS software (SAS Institute Inc., version 9.4).

## Results

### Description of the sample

A total of 33,384 participants of the NutriNet-Santé cohort study completed the Org-FFQ. Among these participants 2097 underreporters and overreporters were excluded, as well as 2320 individuals with missing covariates (which are required to assess inappropriate energy intake), and 722 participants residing in overseas territories. From those 28,245 individuals, 27,843 completed the CFC-12. Then, 209 participants who presented an acquiescence bias (agreeing to all questions without consideration of reversed items) in answers of the CFC-12 were excluded, leaving 27,634 participants in the final analysis. A total of 51,394 participants from the NutriNet-Santé study completed the CFC-12 questionnaires. Compared to excluded participants, the 27,634 participants in the final analysis were older (53.2 ± 14.1 years old for included participants vs 47.1 ± 14.5 years old for excluded participants, *p* < .0001), more often men (25.6% vs. 20.5%, *p* < .0001), had less often a university education (34.6% vs. 40.5%, *p* < .0001). In average, included participants had a lower CFC score (40.5 ± 7.1 vs. 40.9 ± 6.9, *p* < .0001). This marginal difference was likely to be significant due to the large sample size.

Table 1 shows characteristics of the sample according to the 4 categories determined by quartiles of the CFC-12. Overall, apart from energy intake, there was a significant linear trend between every variable analyzed and the categories of the CFC-12 (all *P* < .0001). Compared to Q1, participants in higher categories of CFC consumed more organic food (overall), were younger, were less often women, had more often a high level of education, were less often unemployed, employee, manual worker or retired and were more often student, self-employed, farmer, from managerial staff or intellectual professions, had more often a high monthly income per household, lived more often in large urban units, had a lower BMI, and had a higher mPNNS-GS.

**Table 1:** Individuals characteristics of the participants of the according to categories of the CFC

	All	Quartiles of Consideration of Future Consequences				*P* ^a^
		Q1	Q2	Q3	Q4	
		< 36	36–40	41–46	> 46	
n	27,634	6670	7107	6964	6893	
%	100	24.1	25.7	25.2	24.9	
CFC-12 (12–60)	40.5 ± 7.1	31.3 ± 3.7	38.0 ± 1.4	43.0 ± 1.4	49.4 ± 3.0	
Total organic food intake (g/d)	760.3 ± 799.7	625.8 ± 738.5	717.3 ± 779.5	775.0 ± 789.4	920.1 ± 857.4	< .0001
Age (years)^b^	53.2 ± 14.1	55.9 ± 13.2	54.6 ± 13.8	51.6 ± 14.2	50.5 ± 14.5	< .0001
Gender (%)						< .0001
Women	74.4	76.0	74.9	74.0	72.7	
Men	25.6	24.0	25.1	26.0	27.3	
Education level (%)						< .0001
Primary	2.8	5.5	3.2	1.7	1.0	
Secondary	32.9	46.6	38.2	27.2	20.1	
Undergraduate	29.6	27.1	30.7	31.5	29.1	
Postgraduate	34.6	20.8	27.8	39.7	49.8	
Occupational status (%)						< .0001
Unemployed	8.8	9.9	8.6	8.1	8.6	
Student	1.2	0.6	0.9	1.3	2.0	
Self-employed, farmer	1.7	1.5	1.6	1.7	2.2	
Employee, manual worker	14.2	17.2	14.8	13.8	11.2	
Intermediate professions	13.8	11.2	13.7	15.9	14.1	
Managerial staff, intellectual profession	21.0	12.5	16.9	24.8	29.4	
Retired	39.3	47.0	43.5	34.5	32.4	
Monthly income (%)^c^						< .0001
< 1200€	10.1	12.2	10.1	8.8	9.3	
1200–1799€	20.9	23.0	21.3	20.6	18.7	
1800–2299€	15.3	16.2	16.3	14.8	13.9	
2300–2699€	10.3	10.1	10.3	10.6	10.2	
2700–3699€	17.7	14.3	17.1	18.6	20.9	
> 3700€	13.2	9.1	11.4	14.8	17.2	
Unwilling to answer	12.3	14.9	13.4	11.5	9.5	
Missing data	0.2	0.2	0.2	0.3	0.3	
Place of residence (%)						< .0001
Rural community	22.0	22.7	22.5	21.3	21.5	
Urban unit with a population < 20,000 inhabitants	15.5	16.8	16.0	14.6	14.4	
Urban unit with a population between 20,000 and 200,000 inhabitants	18.0	18.7	18.4	18.0	17.0	
Urban unit with a population > 200,000 inhabitants	44.5	41.8	43.1	46.0	47.1	
BMI (kg/m^2^)^b^	24.2 ± 4.5	25.0 ± 5.0	24.4 ± 4.6	24.0 ± 4.4	23.3 ± 4.0	< .0001
Energy intake (kcal/d)^b^	1994 ± 629	2017 ± 646	1982 ± 632	1975 ± 615	2002 ± 620	.32
mPNNS-GS^b^	8.5 ± 1.8	8.4 ± 1.8	8.5 ± 1.8	8.5 ± 1.7	8.6 ± 1.7	< .0001

mPNNS-GS, modified Programme National Nutrition Santé Guideline Score

^a^*p*-value based on linear trend for continuous variables or Mantel-Haenszel chi-square test for categorical variables (corrected for multiple testing with a Hochberg procedure)

^b^Mean ± SD

^c^Monthly income represents the household income per month calculated by consumption unit (CU). The number of people of the household was converted into a number of CU according to a weighting system: one CU is attributed for the first adult in the household, 0.5 for other persons aged 14 or older and 0.3 for children under 14 [27]

Table 2 shows the percentages of organic food consumers in the 17 food groups according to CFC-12 categories. A significant linear trend was observed for every food group (all *P* < .0001).

**Table 2:** Percentages of organic food consumers by food group according to quartiles of the CFC

	All		Quartiles of consideration of future consequences				*P* ^a^
Food groups (%)	n	%	Q1	Q2	Q3	Q4	
All	27,634	93.5	90.4	92.8	94.6	96.0	< .0001
Fruits and vegetables (including juices and soups)	27,618	83.0	77.3	81.7	84.9	87.8	< .0001
Seafood	26,579	50.8	45.3	50.7	52.7	54.2	< .0001
Meat, poultry, and processed meat	26,550	73.3	68.2	72.3	75.6	77.1	< .0001
Eggs	26,494	76.6	71.2	75.4	78.5	81.5	< .0001
Dairy products	27,177	69.7	62.6	68.1	72.1	75.8	< .0001
Starchy refined foods	27,603	77.5	70.4	75.6	80.1	83.6	< .0001
Whole-grain products	22,527	71.4	63.9	69.6	73.8	77.2	< .0001
Legumes	24,811	55.0	47.2	52.5	57.0	62.5	< .0001
Fats (oil, butter, and margarine)	27,537	71.3	64.4	69.9	73.1	77.6	< .0001
Fatty sweets (including cake, chocolate, ice cream, and pancakes)	27,501	69.1	61.1	67.2	72.1	75.7	< .0001
Non-fatty sweets (including honey, jelly, sugar, and candy)	26,296	69.7	63.1	68.2	71.9	75.3	< .0001
Fast food	26,838	53.0	45.1	51.4	56.0	59.2	< .0001
Snacks (including chips and salted biscuits)	25,902	47.0	39.1	45.5	49.1	54.0	< .0001
Dressings and sauces	27,126	55.7	48.7	54.5	57.7	61.5	< .0001
Dairy products and meat substitutes (including soya-based products)	11,382	85.7	82.0	84.8	86.5	88.3	< .0001
Non-alcoholic beverages	27,552	67.1	58.9	64.7	69.8	74.7	< .0001
Alcoholic beverages	25,417	58.7	50.5	56.6	61.5	65.7	< .0001

^a^*p*-value is based on χ^2^ test and adjusted for multiple testing with a Hochberg procedure

### Association between consideration of future consequences and organic food consumption

Table 3 presents the results of logistic regression models between categories of the CFC-12 and organic food consumption and shows significant linear trends for most food groups. Overall, future oriented participants were more likely to consume organic foods. In particular, compared to Q1, participants in higher categories of the CFC-12 were more likely to consume organic: fruits and vegetables, eggs, dairy products, starchy refined foods, whole-grain products, legumes, fats, fatty sweets, non-fatty sweets, alcoholic beverages, non-alcoholic beverages, fast food, snacks, and dressing and sauces. Three food groups did not present a significant linear trend: seafood, meat, poultry and processed meat, and dairy products and meat substitutes. Comparing Q4 vs. Q1, the strongest associations were found for starchy refined foods (OR = 1.78, 95% CI: 1.63, 1.94), fruits and vegetables (OR = 1.74, 95% CI: 1.58, 1.92), and non-alcoholic beverages (OR = 1.72, 95% CI: 1.59, 1.85).

**Table 3:** Logistic regression models between CFC and the likelihood to consume organic foods

		Quartiles of consideration of future consequences^a^				
		Q1	Q2	Q3	Q4	*P* ^b^
Food groups	n	Ref.	OR (95% CI)	OR (95% CI)	OR (95% CI)	
All	27,634	1	1.24 (1.09, 1.40)	1.51 (1.32, 1.74)	1.88 (1.62, 2.20)	.0043
Fruits and vegetables (including juices and soups)	27,618	1	1.22 (1.12, 1.33)	1.46 (1.33, 1.60)	1.74 (1.58, 1.92)	< .0001
Seafood	26,579	1	1.20 (1.12, 1.29)	1.26 (1.18, 1.36)	1.30 (1.21, 1.39)	.32
Meat, poultry, and processed meat	26,550	1	1.15 (1.07, 1.24)	1.31 (1.21, 1.42)	1.34 (1.23, 1.46)	.08
Eggs	26,494	1	1.18 (1.09, 1.27)	1.34 (1.23, 1.46)	1.53 (1.40, 1.67)	.0008
Dairy products	27,177	1	1.20 (1.11, 1.29)	1.36 (1.26, 1.47)	1.55 (1.43, 1.68)	.0003
Starchy refined foods	27,603	1	1.22 (1.13, 1.32)	1.49 (1.38, 1.62)	1.78 (1.63, 1.94)	< .0001
Whole-grain products	22,527	1	1.25 (1.15, 1.36)	1.49 (1.37, 1.62)	1.66 (1.52, 1.82)	.0002
Legumes	24,811	1	1.18 (1.09, 1.27)	1.35 (1.25, 1.46)	1.55 (1.44, 1.68)	< .0001
Fats (oil, butter, and margarine)	27,537	1	1.22 (1.13, 1.31)	1.38 (1.28, 1.49)	1.67 (1.54, 1.81)	< .0001
Fatty sweets (including cake, chocolate, ice cream, and pancakes)	27,501	1	1.23 (1.15, 1.33)	1.48 (1.37, 1.60)	1.67 (1.55, 1.81)	< .0001
Non-fatty sweets (including honey, jelly, sugar, and candy)	26,296	1	1.22 (1.13, 1.31)	1.42 (1.32, 1.54)	1.62 (1.49, 1.75)	< .0001
Fast food	26,838	1	1.24 (1.16, 1.33)	1.44 (1.34, 1.55)	1.57 (1.46, 1.69)	.0002
Snacks (including chips and salted biscuits)	25,902	1	1.26 (1.17, 1.36)	1.42 (1.32, 1.53)	1.63 (1.51, 1.76)	< .0001
Dressings and sauces	27,126	1	1.23 (1.15, 1.32)	1.40 (1.30, 1.50)	1.58 (1.47, 1.70)	< .0001
Dairy products and meat substitutes (including soya-based products)	11,382	1	1.19 (1.02, 1.38)	1.36 (1.17, 1.59)	1.51 (1.28, 1.77)	.30
Non-alcoholic beverages	27,552	1	1.20 (1.12, 1.29)	1.44 (1.33, 1.55)	1.72 (1.59, 1.85)	< .0001
Alcoholic beverages	25,417	1	1.21 (1.13, 1.30)	1.40 (1.30, 1.51)	1.59 (1.48, 1.72)	< .0001

^a^Model 2: model adjusted on age, gender, education level, occupational status, monthly income per household unit, place of residence, BMI, energy intake, mPNNS-GS (modified Programme National Nutrition Santé Guideline Score), and total food intake of the food group considered in the model

^b^adjusted *p*-value for trend (correction for multiple testing with a Hochberg procedure)

Table 4 focuses on organic consumers and shows the proportions of organic food consumed out of the total intake across categories of the CFC-12. The ratio of total organic food intakes out of total intakes significantly increased from 20.39% (Q1) to 27.12% (Q4) with a relative difference of 33%. There was a significant increase of the proportion of organic food consumed (on total intakes) for almost every group: fruits and vegetables, seafood, meat, poultry and processed meat, eggs, dairy products, starchy refined foods, whole-grain products, legumes, fats, fatty sweets, non-fatty sweets, alcoholic beverages, non-alcoholic beverages, fast food, snacks, dressings and sauces, and dairy products and meat substitutes. Seafood was the only food group without a statistical significant *p*-value for linear trend. The highest relative differences between Q4 and Q1 were observed for starchy refined foods (22%), and non-alcoholic beverages (21%); whereas the lowest relative differences were observed for dairy products and meat substitutes (6%), and eggs (7%).

**Table 4:** Adjusted proportions of organic food intake out of total food intake, by food group, among consumers of organic foods

		Categories of consideration of future consequences								Relative difference between Q4-Q1 (%)	*P* ^a^
	n	Q1		Q2		Q3		Q4			
Food groups		mean^b^	SE	mean^b^	SE	mean^b^	SE	mean^b^	SE		
All	25,828	20.39	0.26	22.40	0.25	23.89	0.25	27.12	0.25	33	< .0001
Fruits and vegetables (including juices and soups)	22,921	37.57	0.40	39.56	0.37	41.09	0.37	44.64	0.37	19	< .0001
Seafood	13,491	31.92	0.46	31.94	0.41	31.79	0.41	33.41	0.42	5	0.13
Meat, poultry, and processed meat	19,464	29.34	0.38	30.52	0.35	30.48	0.35	33.16	0.36	13	< .0001
Eggs	20,307	69.32	0.44	69.83	0.41	71.06	0.40	74.22	0.41	7	< .0001
Dairy products	18,936	36.50	0.47	38.16	0.43	40.24	0.43	43.59	0.43	19	< .0001
Starchy refined foods	21,385	34.05	0.42	35.86	0.39	37.47	0.38	41.41	0.38	22	< .0001
Whole-grain products	16,089	53.28	0.54	53.67	0.48	55.56	0.46	59.96	0.46	13	< .0001
Legumes	13,646	56.23	0.57	57.04	0.51	58.38	0.49	62.47	0.47	11	< .0001
Fats (oil, butter, and margarine)	19,635	47.52	0.49	48.40	0.45	51.31	0.44	55.35	0.44	16	< .0001
Fatty sweets (including cake, chocolate, ice cream, and pancakes)	19,000	29.06	0.41	30.56	0.37	31.88	0.36	34.72	0.36	19	< .0001
Non-fatty sweets (including honey, jelly, sugar, and candy)	18,336	52.19	0.49	53.12	0.45	55.05	0.44	58.63	0.44	12	< .0001
Fast food	14,224	32.28	0.49	33.67	0.44	34.09	0.42	37.94	0.42	18	< .0001
Snacks (including chips and salted biscuits)	12,183	41.84	0.57	41.64	0.50	43.74	0.49	47.81	0.47	14	< .0001
Dressings and sauces	15,099	38.79	0.54	40.06	0.49	41.64	0.48	46.18	0.47	19	< .0001
Dairy products and meat substitutes (including soya-based products)	9758	77.45	0.66	77.87	0.60	79.00	0.56	82.39	0.53	6	< .0001
Non-alcoholic beverages	18,487	17.74	0.29	19.21	0.26	19.53	0.26	21.40	0.25	21	< .0001
Alcoholic beverages	14,908	28.41	0.42	28.54	0.38	28.80	0.37	31.20	0.37	10	< .0001

*SE* standard error

^a^adjusted *p*-value for trend (correction for multiple testing with a Hochberg procedure)

^b^adjusted for age, gender, education level, occupational status, monthly income per household unit, place of residence, BMI, energy intake, mPNNS-GS (modified Programme National Nutrition Santé Guideline Score), and total food intake of the food group considered in the model

## Discussion

Overall, analyses performed in this study showed higher organic food consumption among future oriented individuals compared to less future oriented participants independently of socioeconomic, lifestyle and dietary characteristics. First, individuals with a high consideration of future consequences were found more likely to eat organic foods. Then, among organic food consumers, future oriented individuals were also found in average to have a higher contribution of organic foods in their diet.

### Characteristics of future oriented individuals

Our results supported previous data of the literature indicating that future oriented individuals were younger [29], had more often a high education level [31], and had a lower BMI [12, 29], compared with less future oriented individuals.

### Association between consideration of future consequences and organic food consumption

Overall, results of the association between consideration of future consequences and organic food consumption showed that future oriented individuals were more likely to consume organic food. Moreover, when considering consumers of organic food groups specifically, the more participants were future oriented, the higher was the average contribution of organic foods in their diet (for almost every food group considered). Therefore, these analyses, focusing either on the whole sample or on organic food consumers specifically, both show a link between CFC and organic food intake. No significant interaction was found between gender and CFC in our study, suggesting no moderation effect of gender on the relationship between CFC and organic food intakes. To our knowledge, our study is the first to take into account the CFC-12 or any other measure of time perspective to assess the likelihood of organic food consumption. One study showed evidence that preferences for food products with an organic logo varied according to the level of future orientation [20].

Although very little data is available on association between psychological traits and organic consumption, many studies have investigated how psychological traits influence motives behind organic food choices. A large list of motives has been found to predict organic food intake, such as environmental and ethics aspects [2–7, 32, 33], or health, food safety and sensory aspects [2, 4, 6, 8, 9, 32, 33]. Broadly, these motives could be divided into two categories: environmental concerns (considered as altruistic motives), and individual concerns such as health (self-centered motives) [34]. Future time perspective has been shown to lead to a pro-environmental behavior [16] and to more health oriented behaviors [11, 12, 14, 15]. Consideration of future consequences could therefore be a psychological construct predicting organic food consumption through altruistic or self-centered motives (or both), which could explain the higher proportion of organic food consumers among future oriented participants. Significant linear trends showing increases of proportions of organic food intakes among organic food consumers across categories of the CFC strengthened this hypothesis.

### Food group differences

Future oriented individuals were more likely to consume organic foods, but strengths of the association varied depending on the food group. The strongest associations were found for starchy refined foods, fruits and vegetables, and non-alcoholic beverages. No associations were found for seafood, meat, poultry and processed meat, and dairy products and meat substitutes. Specific factors could play an important role and weaken the relationship between time perspective and the consumption of these organic food groups. For example, constraints like price or origin of the product represent important factors in food choices and purchases [35]. Organic products with the highest differences in price between the organic and the conventional version such as meat, poultry and processed meat [36] could be consumed less because the price constraint would be too important [37]. Moreover, differences in the availability across products could also explain these different associations [38]. For example, less available products could be less purchased and less likely to be consumed. Some studies showed that fruits and vegetables intake in the diet was the most popular organic foods in proportion, whereas meat products and fishes were the least popular products [5, 39]. Our results also suggest that the strongest associations were found in the food groups which show or which are thought to have the greatest benefits for the environment or health (or both) when consumed in their organic version compared to their conventional versions, and inversely for some of the weakest associations. Maximum residue level exceedances are higher in conventionally produced products compared to organic products [40].

In addition, among consumers of organic food products, the highest relative differences in proportions of organic intakes between Q4 and Q1 were found for starchy refined foods, non-alcoholic beverages, while the weakest variation were found for dairy products and meat substitutes, and eggs. The strength of the associations found for this analysis was similar to previous results. Similarly, observed differences between food groups could be explained by constraints regarding price, access, and environment, even among organic food consumers [38].

### Strengths and limitations

The main strength of this study is its large sample size with subjects of various socio-demographic characteristics which allows controlling for confounding factors while keeping a reasonable statistical power. Yet, other potential confounders were not taken into account, such as environmental factors and the availability of organic food. The second strength of this study was the use of a semi-quantitative food frequency questionnaire of 264 items which allowed a reliable estimation of usual diet over the previous year for conventional and organic intakes, despite a possibility of overestimation of intakes. The two complementary analyses, on the whole sample, and on organic food consumers specifically, bring more confidence on the relationship between consideration of future consequences and organic food intake. Questions concerning frequency of organic food consumption were not validated and could have led to misestimate the percentages of organic food consumer or proportions of organic food consumption in the diet. However, the estimation of organic food consumers was not substantially modified in a sensitivity analysis assessing the robustness of the scale [24]. In addition, considering that the completion of the Org-FFQ was optional and the long set of items of the questionnaire, participants with more sustainable food concerns could be more likely to complete it compared to other participants of the cohort. Finally, a high proportion of women and of individuals with a high level of education was included in our analysis, whom have been shown to have greater sustainable consumption [41, 42].

A selection bias could be present because of the method used to recruit participants, which is based on volunteering. The NutriNet-Santé being a cohort focusing on nutrition, its participants are more likely to be interested in nutrition-related issues. Consequently, our subjects may have high health awareness, and a high interest toward organic food and sustainability issues compared to the general population; meaning that percentages of organic food consumers were probably not representative of the general population. Another limitation of this study is its design, which is a cross-sectional analysis within a cohort, and thus does not allow us to assess causality. Moreover, all collected data were self-reported which could have led to measurement errors. The CFC questionnaire has been widely used with health and environmental outcomes. Recent studies reported a two-factor structure of the CFC, distinguishing immediate and future subscales [43]. Even though there is no consensus on the use of the CFC [44], a two-dimension analysis could have added another perspective in our interpretation of the results.

## Conclusions

This study showed that consideration of future consequences could be considered as a construct associated with consumption of organic food. For the majority of the assessed food groups, participants with the highest future orientation were more often consumers of organic foods, and when consuming these foods consumed a higher quantity of them. More generally, time perspective could be a personality trait predicting environmental and health concerns, and could be a key psychological factor influencing dietary behaviors and in particular organic food intake. These findings could explain the cognitive process underlying organic food choices and show the importance to take individual’s psychological factors into account regarding overall food choices. Promoting the importance of future outcomes and long-term benefits could represent an approach of public health programs aiming at encouraging intake of organic food or more generally health promotion and chronic disease prevention.

## Additional file

Additional file 1: Participant flow chart from the NutriNet-Santé cohort study (2014) included in the current analysis. (DOCX 48 kb)

## Abbreviations

CFC: Consideration of future consequences
CNIL: Commission Nationale Informatique et Libertés
CU: Consumption unit
IRB: International Research Board of the French Institute for Health and Medical Research
mPNNS-GS: Modified Programme National Nutrition Santé Guidelines Score
Org-FFQ: Organic food frequency questionnaire
