# Supplementary material for: Association between time perspective and organic food consumption in a large sample of adults
Source: Nutr J. 2018 Jan 5;17:1. doi: 10.1186/s12937-017-0311-0 (PMC5755405; doi:10.1186/s12937-017-0311-0)
Supplement: Additional file 1: — Participant flow chart from the NutriNet-Santé cohort study (2014) included in the current analysis. (DOCX 48 kb) [file 12937_2017_311_MOESM1_ESM.docx]

*Participant flow chart from the NutriNet-Santé cohort study (2014) included in the current analysis.*

156,623 participants were included in the NutriNet-Santé study (June 2014)

33,384 participants completed the Org-FFQ questionnaire

4,417 with inappropriate energy intake (under reporters & over reporters)

28,967 participants with appropriate energy intake

722 participants residing in overseas territories were excluded

28,245 not residing in overseas territories

From the 28,245 remaining participant, 402 did not completed the CFC-12 questionnaire

27,843 participants completed the Org-FFQ and the CFC-12 questionnaire

51,394 participants completed the the CFC-12 questionnaire

209 participants who presented an acquiescence bias were excluded

27,634 participants were considered for the final analysis (7,077 men and 20,557 women)
